# Hepatitis A notifications in the EU/EEA, 2010–2019: what can we learn from case reporting to the European Surveillance System?

**DOI:** 10.2807/1560-7917.ES.2023.28.19.2200575

**Published:** 2023-05-11

**Authors:** Ettore Severi, Lara Tavoschi, Paloma Carrillo-Santisteve, Therese Westrell, Gaetano Marrone, Johan Giesecke, Pierluigi Lopalco

**Affiliations:** 1 European Centre for Disease Prevention and Control (ECDC), Stockholm, Sweden; 2 Karolinska Institutet, Department of Medical Epidemiology and Biostatistics, Stockholm, Sweden; 3 University of Pisa, Department of Translational Research and New Technologies in Medicine and Surgery, Pisa, Italy; 4 Health Directorate, Office de la Naissance et de l'Enfance, Brussels, Belgium; 5 University of Salento, Department of Biological and Environmental Science and Technology, Lecce, Italy

**Keywords:** Infectious diseases, hepatitis A, incidence, European Union, surveillance, outbreaks

## Abstract

**Background:**

European Union/European Economic Area (EU/EEA) countries annually report hepatitis A (HepA) notifications to The European Surveillance System (TESSy).

**Aim:**

To describe EU/EEA HepA notifications from 2010 to 2019 and identify infection drivers and surveillance improvements.

**Methods:**

We analysed demographic, clinical and transmission information of HepA confirmed cases from TESSy. We stratified countries by population susceptibility profile and performed time-series analysis to describe trends in notification rates, sex distribution and travel history.

**Results:**

Twenty-nine EU/EEA countries reported 139,793 HepA cases. Six eastern EU countries reported > 60% of these cases. EU/EEA notification rate during the study period was 3.2 cases per 100,000 population (range 2.7–5.6). Notifications peaked in 2014 and 2017, with marked differences in case demographic characteristics. Notification trends varied across different country susceptibility groups. In 2017, the proportion of males (74%) and case median age (31 years) increased steeply, while no changes occurred in 2014. Travel history showed seasonal case peaks following the summer. More than 47,000 hospitalisations were reported. Annual case fatality was < 0.2% for all years. Information on travel history, hospitalisation, death and mode of transmission was suboptimal.

**Discussion:**

Apart from some countries in its east, the EU/EEA is characterised by low HepA incidence baseline and susceptible to recurrent large cross-border outbreaks. Analysis of European surveillance data highlighted the need for stronger prevention policies for eastern EU countries, men who have sex with men and travellers. Improving surveillance data-quality will enhance knowledge on food-borne, and travel-related exposures to inform more effective and tailored regional prevention policies.

**Key public health message**

**What did you want to address in this study?**

We wanted to describe the results from data routinely collected for hepatitis A in European Union/European Economic Area (EU/EEA) countries and show which areas and population groups are most vulnerable to hepatitis A virus infection. We also wanted to suggest improvements that could make hepatitis A surveillance data more useful for public health action.

**What have we learnt from this study?**

We learnt that the epidemiology of hepatitis A has different features in different EU/EEA countries. Eastern EU countries had the highest number of infections, which often affected children or young adults. Numbers were lower in other EU/EEA countries where the population is susceptible to cross-border outbreaks linked to food or at-risk-sexual practices, such as those that happened in 2013–2014 and 2017, respectively.

**What are the implications of your findings for public health?**

Hepatitis A is still a public health issue in the EU/EEA. Policy makers could consider enhancing food safety, strengthening risk communication and vaccinating risk groups, as per WHO recommendations. The European surveillance of hepatitis A could be improved to strengthen planning of preventive or control measures. Rapid detection, alert and cross-border information-sharing are essential tools to limit the extent of hepatitis A outbreaks.

## Introduction

Hepatitis A (HepA) is an acute viral infection caused by the hepatitis A virus (HAV). Virus transmission occurs mainly through the faecal-oral route via person-to-person contact, ingestion of contaminated food or water or very rarely through infected blood [1-3]. Hepatitis A virus infection is mostly asymptomatic in children under 6 years of age. The proportion of symptomatic cases and the severity of infection increases with age. Severe outcome such as liver failure or death, although rare, occur more frequently in patients older than 50 years of age or in persons with underlying chronic liver disease [2,4].

As a result of improved hygiene, sanitation, socioeconomic conditions and increased availability of vaccines, HAV incidence has declined in the European Union/European Economic Area (EU/EEA). This transition has occurred at different times in different countries, e.g. several decades ago in Nordic countries, while recently in eastern EU countries [5]. At the same time, the proportion of the EU/EEA population susceptible to HAV has substantially increased [5,6]. Outbreaks occur not only in groups traditionally at increased risk of HAV infection such as travellers, men who have sex with men (MSM), people who inject drugs (PWID) and underserved population groups and their close contacts, but also in the general population when exposed to contaminated food distributed within the EU single market [7-13].

On an annual basis, EU/EEA countries report surveillance data on HepA to The European Surveillance System (TESSy) hosted by the European Centre for Disease Prevention and Control (ECDC), according to Decision 1082/2013/EU on serious cross-border threats to health [14]. Surveillance should provide information that can serve public health action. At the EU/EEA level, surveillance objectives are to monitor disease trends, identify groups at risk, infection sources and modes of transmission over time and across the EU/EEA, evaluate and monitor programmes and interventions, assess the burden of communicable diseases and identify needs for research [15]. Specifically for HepA, timely detection of cross-border outbreaks do not rely on TESSy but on the Epidemic Intelligence Information System for Food- and Waterborne diseases (EPIS-FWD, which in 2020 became EpiPulse) [16]. In line with the EU/EEA surveillance objectives, data collected should be of high quality.

Using data from TESSy, we aim to describe HepA notifications in the EU/EEA from 2010 to 2019 and discuss HAV infection drivers and areas for possible surveillance improvements.

## Methods

### Data source and study population

We used TESSy data containing HepA notifications from 2010 to 2019, as reported by 29 EU/EEA countries. The study population included the whole EU/EEA population, excluding Liechtenstein, which does not report HepA data to TESSy, and the United Kingdom (UK), which started the process of leaving the EU in 2016.

All 29 EU/EEA countries reported HepA information from comprehensive surveillance systems except Belgium, which used a sentinel surveillance system and had to be excluded from the annual notification rate calculation. All countries reported case-based data for the whole study period, except for three countries that reported aggregated data (Belgium from 2015 to 2019, Bulgaria from 2010 to 2019 and Poland in 2010). Data from Belgium and Bulgaria were excluded from all analyses, except the calculation of the Bulgarian annual notification rates. Data for Poland were included as 2010 granularity was deemed sufficient. Case-based data included information on demographic (age and sex), clinical (hospitalisation and death), diagnostic (dates of onset, diagnosis and receipt at the reference laboratory), travel history and mode of transmission (food, healthcare-associated, person-to-person, PWID, recreational water, sex, transfusion, other or unknown exposure). Every year, each national authority reports a dataset to TESSy containing information on the previous reporting year. After data cleaning, management and analysis, the data are disseminated by ECDC through annual epidemiological reports. Since 2015, data are also published in the Surveillance Atlas of Infectious Diseases [17]. We extracted data from TESSy on 25 January 2022.

### Case definition

We used the EU surveillance case definition for confirmed HepA cases [18]. A case was any person meeting both clinical (fever, jaundice and/or elevated serum aminotransferase levels with a discrete onset of symptoms) and laboratory (detection of HAV nucleic acid in serum or stool, anti-HAV IgM response and/or detection of HAV antigen in stool) criteria. In countries where surveillance does not capture clinical symptoms, laboratory criteria are sufficient to define a confirmed case.

### Descriptive statistics and variables

We described notifications of HepA confirmed cases by year and country. We used Eurostat estimates on mid-year annual country populations to calculate notification rates [19]. Notification rates were not calculated for Belgium because reporting from sentinel surveillance and Belgium was not included in the EU/EEA notification rate calculation.

We grouped countries into four groups based on their population susceptibility to HAV infection based on our latest available information [5]: (i) low susceptibility  (< 30% seronegative at age 30 or 50 years); (ii) moderate susceptibility (30–50% seronegative at age 30 years and < 30% seronegative at age 50 years); (iii) high susceptibility (50–70% seronegative at age 30 years and 30–50% seronegative at age 50 years); (iv) very high susceptibility ( > 70% seronegative at age 30 and > 50% seronegative at age 50 years) as per previously reported methodology [5]. We assigned Hungary and Latvia, for which susceptibility profiles were not available, to the low and moderate susceptibility groups, respectively, based on their notification rates from 2010 to 2019.

For all included EU/EEA countries and for each susceptibility group, we calculated case median age at infection and proportion of male cases by year. For those countries with data completeness ≥ 80%, we also described information on patient hospitalisation and death. Hospitalisation was presented as the proportion of hospitalised cases among all patients during the same year, while case fatality ratio was defined as deaths every 100 cases among all cases and among those ≥ 50 years old (case fatality 50 +). We also described reported routes of transmission.

In the descriptive time series analyses, we grouped cases by ’month of statistics’, a field in TESSy, (in order of priority: (i) month of onset; (ii) month of diagnosis or (iii) month of receipt at the reference laboratory). We excluded cases either because the country reported aggregated data without information on month of statistics, or because case information was missing or reported as unknown. We used time series analysis to depict the monthly frequency of notifications, the male and female notification rates per 100,000 population and the frequency of notifications stratified by travel history. Each time series was complemented by a 12-month moving average and linear monthly notification trend, both for all EU/EEA countries and country susceptibility groups.

## Results

**Figure 1:**
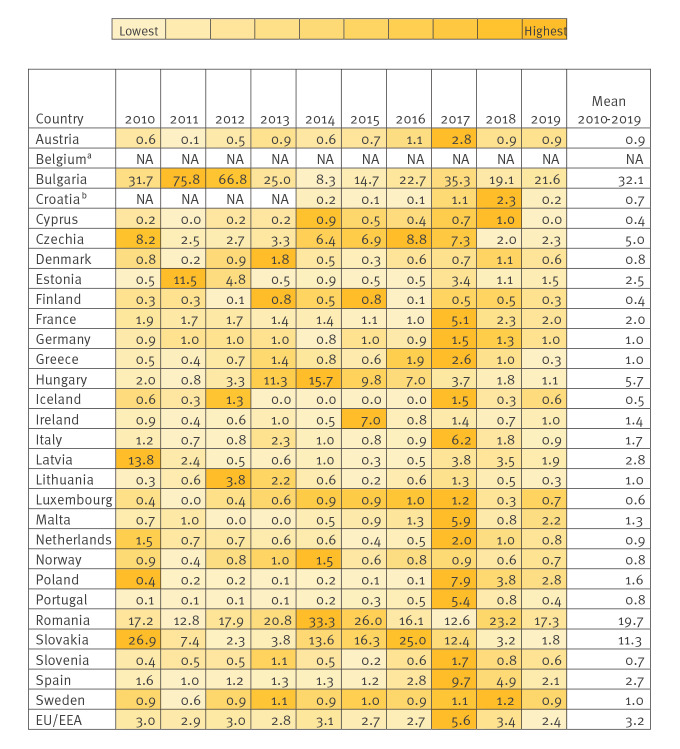
Hepatitis A notification rates (cases per 100,000 population) by country and reporting year, EU/EEA countries, 2010–2019 (n = 139,793)

We identified 139,793 HepA confirmed cases reported by 29 EU/EEA countries to TESSy from 2010 to 2019. Of all cases, 45% (n = 62,525) were reported by Bulgaria and Romania. The notification rates of confirmed cases ranged from 0 per 100,000 population in countries reporting no cases for 1 year (Cyprus in 2011 and 2019, Iceland 2013–2016, Luxembourg in 2011 and Malta 2012–2013) to 75.8 cases per 100,000 population in Bulgaria in 2011 (Figure 1). At the national level, the mean notification rate during the study period was ≤ 1 case per 100,000 population in 15 EU/EEA countries and ≥ 5 cases per 100,000 population in five EU countries (Bulgaria, Czechia, Hungary, Romania and Slovakia). The EU/EEA notification rate for the whole period was 3.2 cases per 100,000 population, ranging from 2.4 (2019) to 5.6 (2017).

EU/EEA: European Union/European Economic Area; NA: not applicable.

^a^ Data for Belgium marked as NA because reporting from sentinel surveillance.

^b^ Data for Croatia marked as NA before 2014 when they entered the European Union.

Data for Belgium, for the study period, and for Croatia, before 2014, were not included in the calculation of the EU/EEA notification rate.

In each country row, annual notification rates are coloured ranging from light yellow to orange with darker colours indicating increasing notification rate values.

**Figure 2:**
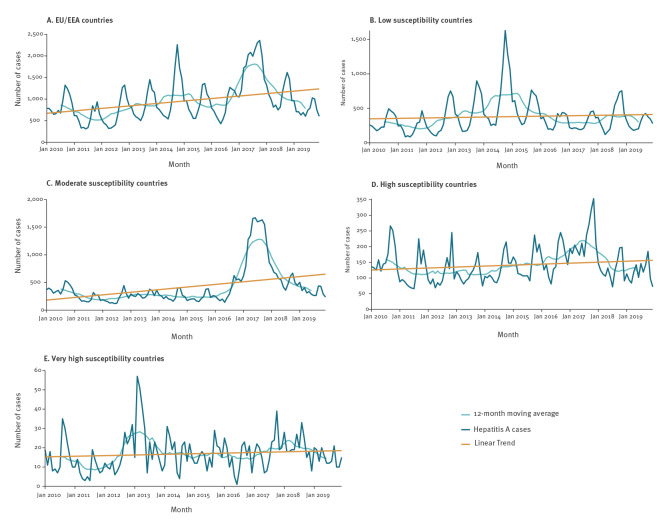
Distribution of hepatitis A notifications, 12-month moving average and linear trend by reporting month, EU/EEA countries, 2010–2019

There was an increasing trend in HepA notifications from 2010 to 2019 (data for all EU/EEA countries except Belgium and Bulgaria). The 12-month moving average showed the lowest frequency of cases occurred in 2011, then increased, culminating in a peak in 2014. This was followed by a decrease in case frequency, then a subsequent increase culminating in 2017 (Figure 2). Seasonal peaks of larger or smaller extent were observed every year at the end of summer and in autumn. The time series by country susceptibility profile showed different dynamics: in low and very high susceptibility countries, the overall trend was rather stable with peaks in notifications in 2014 and 2013, respectively. In moderate susceptibility countries, and to a lesser extent in high susceptibility countries, the overall increasing trend was driven by a large peak in 2017.

EU/EEA: European Union/European Economic Area.

A: all EU/EEA countries except Belgium and Bulgaria.

B: low hepatitis A virus infection susceptibility countries (< 30% seronegative at age 30 or 50 years) include Hungary, Portugal, Romania.

C: moderate hepatitis A virus infection susceptibility countries (30–50% seronegative at age 30 years and < 30% seronegative at age 50 years) include Cyprus, France, Greece, Italy, Lithuania, Latvia, Malta, Poland, Slovenia, Slovakia, Spain.

D: high hepatitis A virus infection susceptibility countries (50–70% seronegative at age 30 years and 30–50% seronegative at age 50 years) include Austria, Croatia, Czechia, Estonia, Germany, Ireland, Luxembourg, the Netherlands.

E: very high hepatitis A virus infection susceptibility countries ( > 70% seronegative at age 30 and > 50% seronegative at age 50 years) include Denmark, Finland, Iceland, Norway, Sweden.

Hepatitis A virus infection susceptibility levels as per previously reported methodology [5].

### Sex and age

For cases with available information (n = 114,311/114,382 with information on month of statistics), males accounted for 59% of cases during the study period, ranging from 53% in 2012 and 2015 to 74% in 2017 (Table). Case median age ranged between 14 and 17 years from 2010 to 2016, doubling to 31 years in 2017 and then decreasing. The distribution of cases by sex and median age showed different dynamics when stratifying countries by HAV infection susceptibility. Low susceptibility countries showed a stable median age of cases (range 11–14 years) and proportion of male cases (range 53–57%). All other susceptibility groups were characterised by a sudden increase in the proportion of male cases in or around 2017 (up to 79% in moderate susceptibility countries). Countries with moderate or very high susceptibility observed a sudden increase in case median age in 2017 (to 33 years old), while high susceptibility countries, which reported the highest median age during the whole study period, observed median age increase (to 40 years old) in 2018 and 2019.

**Table:** Hepatitis A cases’ median age and proportion of males by hepatitis A virus infection susceptibility region and reporting year, EU/EEA countries, 2010–2019

Susceptibility region	Age and sex	Reporting year																				All years 2010–2019	
		2010		2011		2012		2013		2014		2015		2016		2017		2018		2019		Total	Median
		n	%	n	%	n	%	n	%	n	%	n	%	n	%	n	%	n	%	n	%		
Low susceptibility	Median age (years)	11		12		13		13		12		12		12		14		11		11		NA	12
	Females	1,712	46	1,241	46	1,802	46	2,371	45	3,719	45	2,912	47	1,746	44	1,461	43	2,220	46	1,618	46	20,802	46
	Males	1,989	54	1,413	54	2,142	54	2,934	55	4,498	55	3,253	53	2,182	56	1,936	57	2,566	54	1,879	54	24,810	54
Moderate susceptibility	Median age (years)	22		23		19		22		17		18		24		33		31		29		NA	27
	Females	1,844	41	1,114	43	1,164	46	1,578	46	1,422	46	1,318	47	1,553	37	3,246	21	2,560	38	1,855	45	17,654	36
	Males	2,622	59	1,449	57	1,351	54	1,861	54	1,658	54	1,488	53	2,620	63	12,478	79	4,162	62	2,270	55	31,959	64
High susceptibility	Median age (years)	23		30		29		33		33		25		30		33		38		40		NA	31
	Females	939	47	631	46	671	49	665	49	704	46	818	47	831	44	963	35	737	45	668	47	7,627	45
	Males	1,049	53	743	54	687	51	687	51	836	54	932	53	1,048	56	1,786	65	915	55	749	53	9,432	55
Very high susceptibility	Median age (years)	21		16		16		22		27		16		15		32		26		26		NA	22
	Females	89	46	43	41	100	52	154	51	95	44	86	45	79	46	76	33	115	46	89	49	926	46
	Males	105	54	61	59	92	48	146	49	120	56	106	5	94	54	152	67	133	54	92	51	1,101	54
All EU/EEA countries^a^	Median age (years)	16		17		16		17		14		14		17		31		24		22		NA	20
	Females	4,584	44	3,029	45	3,737	47	4,768	46	5,940	46	5,134	47	4,209	41	5,746	26	5,632	42	4,230	46	47,009	41
	Males	5,765	56	3,684	55	4,272	53	5,628	54	7,112	54	5,779	53	5,944	59	16,352	74	7,776	58	4,990	54	67,302	59

EU/EEA: European Union/European Economic Area; NA: not applicable.

^a^ Except Belgium and Bulgaria.

Countries included in the analysis: low HAV infection susceptibility  (< 30% seronegative at age 30 or 50 years) Hungary, Portugal, Romania; moderate HAV infection susceptibility (30–50% seronegative at age 30 years and < 30% seronegative at age 50 years), Cyprus, France, Greece, Italy, Lithuania, Latvia, Malta, Poland, Slovenia, Slovakia, Spain; high HAV infection susceptibility (50–70% seronegative at age 30 years and 30–50% seronegative at age 50 years) Austria, Croatia, Czechia, Estonia, Germany, Ireland, Luxembourg, the Netherlands; very high HAV infection susceptibility ( > 70% seronegative at age 30 and > 50% seronegative at age 50 years) Denmark, Finland, Iceland, Norway, Sweden.

Hepatitis A virus infection susceptibility levels as per previously reported methodology [5].

**Figure 3:**
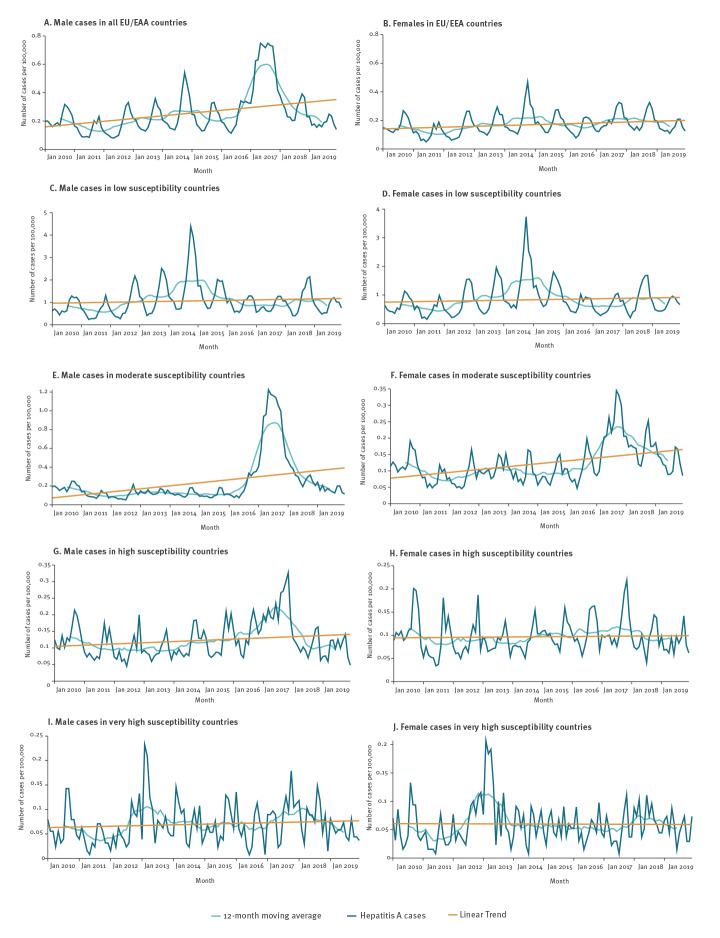
Distribution of hepatitis A notifications by 100,000 population, 12-month moving average and linear trend by reporting month and sex, EU/EEA countries, 2010–2019

Time series analysis stratified by male and female notification rates showed an increasing trend more accentuated in males, which peaked in 2017. The most prominent peak in female notification rates was seen in 2014 (Figure 3, including data for all EU/EEA countries except Belgium and Bulgaria). When stratifying by country susceptibility, male and female rates became more consistent within the same group of countries. In countries at low and very high susceptibility, the highest peak in notification rates in both sexes appeared in 2014, while in moderate and high susceptibility countries notification rates for both sexes peaked in 2017. Such peaks were more pronounced in males than females in all groups except low susceptibility countries.

EU/EEA: European Union/European Economic Area.

^a^ All EU/EEA reporting countries except Belgium and Bulgaria.

^b^ Low hepatitis A virus infection susceptibility countries (< 30% seronegative at age 30 or 50 years: Hungary, Portugal, Romania.

^c^ Moderate hepatitis A virus infection susceptibility countries (30–50% seronegative at age 30 years and < 30% seronegative at age 50 years): Cyprus, France, Greece, Italy, Lithuania, Latvia, Malta, Poland, Slovenia, Slovakia, Spain.

^d^ High hepatitis A virus infection susceptibility countries (50–70% seronegative at age 30 years and 30–50% seronegative at age 50 years): Austria, Croatia, Czechia, Estonia, Germany, Ireland, Luxembourg, the Netherlands.

^e^ Very high hepatitis A virus susceptibility countries ( > 70% seronegative at age 30 and > 50% seronegative at age 50 years): Denmark, Finland, Iceland, Norway, Sweden.

Hepatitis A virus susceptibility levels as per previously reported methodology [5].

### Travel history

**Figure 4:**
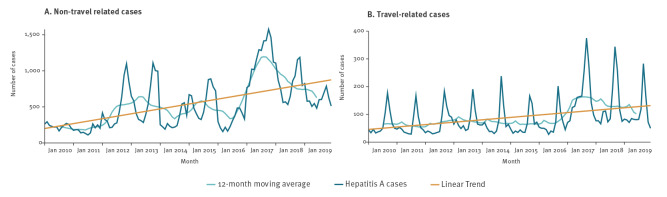
Distribution of hepatitis A notifications, 12-month moving average and linear trend by reporting month and travel-related status, EU/EEA countries, 2010–2019

Information on history of travel during the exposure period was available for 75,195/114,382 cases (65.7% with information on month of statistics), with only 15 countries having ≥ 80% data completeness (Austria, Denmark, Estonia, Finland, France, Greece, Hungary, Latvia, Malta, the Netherlands, Norway, Poland, Portugal, Spain, Sweden). There were 10,657 (14.1%) cases reported as travel-related (Figure 4). Time series analyses stratified by travel history showed a more pronounced linear increase in non-travel related cases and a peak in both travel and non-travel related cases in 2017 and the following years. During the study period, the late summer/early autumn peaks in travel-related cases slightly preceded the peaks in non-travel-related cases.

EU/EEA: European Union/European Economic Area.

### Hospitalisation and death

Information with ≥ 80% completeness  on patient hospitalisation (47,436 hospitalised/52,198 cases) was available for 16 countries (Austria, Cyprus, Denmark, Estonia, Greece, Hungary, Ireland, Lithuania, Luxembourg, Malta, the Netherlands, Norway, Poland, Portugal, Romania and Slovenia), while information with ≥ 80% completeness on death (73 deaths/74,763 cases) was available for 17 countries (Austria, Cyprus, Czechia, Estonia, Germany, Greece, Hungary, Latvia, Lithuania, Luxembourg, Malta, the Netherlands, Poland, Portugal, Romania and Slovakia).

The largest annual peak in hospitalisations was seen in 2014 (8,087), followed by 2017 (6,252) and 2015 (6,049). The years with the highest proportion of hospitalisations among all reported cases were 2012, 2014, 2015 and 2019 (93%). Romania reported 34,446 (72.6%) of all 47,436 hospitalised cases from 2010 to 2019 with close to 100% of cases hospitalised. Excluding data from Romania, the highest number of hospitalised cases was reported in 2017 (3,775), followed by 2018 (1,275), while the highest proportion of cases hospitalised was reported in 2018 (80%).

Germany and Poland reported 20 and 19 of the 73 deaths recorded during the study period, respectively. The years with the most deaths reported for all countries were 2017 (n = 19), 2018 (n = 14) and 2019 (n = 13). For all countries, case fatality ranged from 0.01% in 2010 to 0.18% in 2019, with 2017 and 2018 also showing case fatality substantially higher than seen in previous years (0.13%). Of the 54 patients older than 50 years of age who died from HepA, 33 died between 2017 and 2019, with a case fatality 50 + reaching 0.6% in 2018 and 2019.

### Transmission route

Information on the suspected route of HAV transmission was missing or classified as ‘unknown’ or ‘other’ for 78% of cases (n = 89,789/114,536). Person-to-person transmission and food-borne transmission were reported for 66% (n = 16,389/24,747) and 30% (n = 7,508/24,747) of the remaining cases, respectively. Sexual transmission and injecting drugs-associated transmission were reported for a minority of cases (3% and < 1%, respectively). Among rare exposures associated with transmission, there was one case of animal transmission (plausibly in a laboratory setting, but no additional information was available), four transfusion-associated cases, seven healthcare-associated infections and 14 cases associated with recreational water use.

## Discussion

This study provided an extensive overview of the current epidemiology of HepA in the EU/EEA, highlighting ongoing dynamics and providing up-to-date evidence to inform future policy choices. Almost 140,000 cases of HepA/HAV infection were reported in the EU/EEA from 2010 to 2019. Most cases and the highest notification rates were reported in eastern EU countries, mainly Bulgaria and Romania. During the study period, notification rates oscillated with an overall increasing trend and large peaks in 2014 and, particularly, in 2017, when major cross-border outbreaks were reported [11,13].

In view of such large differences in HepA epidemiology in different EU/EEA countries, when data completeness allowed, we opted for presenting data for groups of countries based on their HAV susceptibility profiles. Such susceptibility indicator was derived from an assessment of both recent and historical HAV infection incidence, allowing for meaningful grouping of countries based on epidemiologic similarities in the past 50 years [5]. Through this approach, it was possible to observe that the increasing EU/EEA trend in notifications occurred mostly in moderate and high susceptibility countries, while low and very high susceptibility countries had a stable trend during the study period. We also opted for not including data for 2020 due to the anomalies related to the COVID-19 pandemic, possibly due to physical distancing policies and travel measures affecting contact patterns and reducing exposure risks in schools, via eating out or travelling abroad.

Stratifying by country, HAV susceptibility profiles showed that the large cross-border outbreaks (2013–2014 food-borne outbreaks and 2017 sexual-transmission outbreak) affected the four country groups differently. On one hand, low and very high susceptibility countries reported large increases in 2013 and 2014, respectively. In very high susceptibility countries, increases were associated with food-borne transmission (associated with different events implicating consumption of strawberries and mixed berries) [12,13,16,20]. It is plausible that this same mode of food-borne transmission also occurred in low susceptibility countries. On the other hand, the 2017 outbreak associated with MSM heavily affected high and moderate susceptibility countries [11].

Similar patterns were observed in the median age and in the proportion of male cases in these different susceptibility groups during the study period. Low susceptibility countries, characterised by higher notification rates throughout the period, reported cases with a median age 10–20 years lower than in other regions. This was expected as a younger mean age of infection tends to be observed in areas with higher incidence of HAV infection [4]. Low susceptibility countries also experienced a more stable proportion of male cases with little apparent change in 2017, confirming that the sexual-transmission outbreak had limited impact compared with other regions. Conversely, the large increase in both male and female reported cases in 2014, which was not associated with an increase in the median age of infection, pointed to a large transmission event, at least in part, driven by food-borne transmission.

The 2017 sudden rise in median age of infection and proportion of male cases suggests that the extent of transmission among MSM sexual networks was the driver of the outbreak not only in countries at moderate and high HAV infection susceptibility, but also in the rest of the EU/EEA countries. In moderate susceptibility countries, the 2017 median age of infection suddenly increased by almost 10 years, with four of five cases being male. In the following 2 years, the proportion of male patients rapidly decreased, while median age declined slowly and remained well above the pre-2017 average. We hypothesise that this could be due to decreased transmission in MSM, coupled by transmission and spill overs in the general community [21].

A similar increase in the median age of infection and proportion of male cases was visible in high and very high susceptibility countries, with some distinct features. In high susceptibility countries, the peak in male cases occurred in 2017 and rapidly normalised in the following 2 years. However, the median age of infection, which was already higher than in the rest of the EU/EEA, increased to 38 and 40 years in 2018 and 2019, respectively. This suggests the large proportion of the older population (> 30 years and > 50 years) susceptible to HAV, with infections spilling over from the MSM network to the general community [5,21]. To a lesser extent, the same was seen in very high susceptibility countries, although their median age of infection was generally lower but increased in 2013 and 2017, illustrating not only the impact of the 2017 sexual-transmission outbreak, but also of the 2013–2014 food-borne outbreaks [13,16,20]. One of these food-borne outbreaks was linked to travel in Egypt [12]. While such outbreaks are well described in the literature, little can be found about the transmission drivers in low susceptibility countries during the same period. The only exception is a publication reporting that the HAV strain associated with the large and prolonged 2013–2014 EU/EEA cross-border food-borne outbreak was retrospectively found to be one of the most frequently circulating virus strains in Bulgaria during the 2012 increase in cases [13,22].

In terms of MSM outbreaks, a male predominance in HepA cases has been previously described, and large European outbreaks affecting the MSM community reported in 2008–2009 and earlier showed a certain degree of HAV endemicity in European MSM. This group was able to sustain transmission of the same HAV strains for much longer than the general population [23,24].

Information on travel history allowed us to observe that HepA cases followed an expected seasonal pattern, with the majority of cases being reported after the holiday season. Late summer and early winter cases are often associated with unvaccinated travellers visiting high endemicity countries during summer and end-of-year holidays [7]. Peaks in travel-related cases slightly precedes those in non-travel related cases, indicating non-travel related secondary transmission following travel-related infections. Even though the World Health Organization (WHO) and most EU/EEA countries recommend vaccinating against hepatitis A for travellers to intermediate and high endemicity countries [7], travel is still a major driver of infections in Europe. Unfortunately, information on travel history is suboptimal in the notifications for many EU/EEA countries, which does not allow for meaningful grouping.

More than 47,000 HepA patients were reported as hospitalised in 16 EU/EEA countries during the study period. This figure is an underestimation of the true number of EU/EEA hospitalisations during the study period as 13 EU/EEA countries, including the most populous, could not be included because hospitalisations were not reported. The analysis of the 2013–2014 food-borne outbreak reported that 70% of cases were hospitalised for a median of 6 days [13]. Hepatitis A has high direct and indirect impact on both patients and health systems [4].

The 73 deaths reported are also likely to be an underestimation. Information on patient deaths is suboptimal in many EU/EEA countries and capturing information on fatal outcome may be challenging in HepA surveillance. Patients may die relatively long after the initial HepA notification and surveillance is not always able to follow up on such information. Thus, it was not surprising to observe a case fatality well below that reported in the literature, both over the whole age stratum and in those older than 50 years of age [2]. However, thanks to high quality free-of-charge universal healthcare offered in most EU/EEA countries and due to the large denominator of reported cases, we expected to see a lower case fatality than reported in the literature. It should also be highlighted that frequency of deaths and the case fatality rate was seen to increase with the increasing age of patients associated with the 2017 outbreak.

Information on the transmission route was poorly completed in the notification data and thus hard to analyse. Although for some sporadic cases it can be challenging to determine the plausible transmission route, the lack of this information for most cases represents a missed opportunity as such information could drive strategic prevention policies.

Our analysis had some limitations. TESSy provides a consistent picture of Hep A notifications during the study period for most EU/EEA countries. However, aggregated case reporting substantially limited the analysis of surveillance data. Although most countries use the European Commission case definitions, differences in the application of such case definitions (e.g. including laboratory-confirmed cases with no information on clinical symptoms) might explain part of the differences between countries. In addition, reported notification rates underestimated the true HAV infection incidence due to under-ascertainment of both the large proportion of asymptomatic infections in children and, to a lesser extent, of infections in population groups at risk of HAV infection who may have limited access to care or laboratory testing (e.g. underserved population groups or migrants) [25].

The overall median age at infection for the whole of the EU/EEA is actually lower than presented in this study since Bulgaria, which reported a high number of cases in children, could not be included in the analysis due to aggregate-based reporting. In addition, since HepA is more severe at an older age, those countries reporting mostly hospitalised cases greatly over-estimated the true median age of infection.

Data from TESSy do not capture information on clinical outcomes, such as liver transplant or length of hospitalisation, which is necessary to understand the full impact on patients and services and therefore plan for suitable and cost-effective interventions.

Finally, in the analysis of low susceptibility countries, Romania reported the majority of cases and is therefore over-represented, while Portugal, according to notification rates in recent years, may have fit better in the moderate or high susceptibility countries group.

Hepatitis A appears still to be a challenge in EU/EEA countries. Even though HepA has low baseline incidence in most EU/EEA countries, high notification rates still occur in some eastern EU countries and all countries remain susceptible to large outbreaks fuelled by food-borne and sexual transmission [26]. Enhanced strategies to prevent disease and outbreaks are needed. Food-borne transmission can be limited by improved food safety and efficient collaboration between public health and food safety authorities. Transmission associated with risky sexual practices can be reduced by improved risk-communication and risk-group vaccination scale-up, particularly among MSM, as per WHO recommendations [27]. Since HepA outbreaks often involve multiple EU/EEA countries, rapid detection, alert and information-sharing within the EU/EEA are essential to rapidly respond and limit the extent of such outbreaks. The systems responsible for rapidly alerting and communicating about outbreaks are the Early Warning and Response System of the European Union (EWRS) and EpiPulse. From 2010 to 2019, most of the inquiries launched by EU/EEA countries in EpiPulse were aimed at alerting and investigating possible cross-border events associated with consumption of contaminated food or transmission in MSM.

Finally, information on whether a case was linked to a known cluster, their HepA vaccination status, and molecular information on viral isolates, currently not reported to TESSy, could help identify the proportion of non-sporadic cases, potential vaccine escape variants and the pattern of virus circulation in the EU. Such information would also support identification of international food-borne outbreaks associated with cases misclassified as sporadic [28-31].

## Conclusions

TESSy remains a useful tool to describe the epidemiology of HepA in EU/EEA countries. However, its ability to plan/evaluate preventive/control measures could be improved by reporting complete high-quality case-based observations on travel history, route of transmission and clinical outcome. Such information would, for example, allow planning and monitoring, or considering policies on increasing vaccination coverage of international travellers or groups at increased risk of severe outcome. It would also allow prioritising responses to events driven by food-borne or sexual transmission.
